# Unsupervised learning of satellite images enhances discovery of late Miocene fossil sites in the Urema Rift, Gorongosa, Mozambique

**DOI:** 10.7717/peerj.11573

**Published:** 2021-06-08

**Authors:** João d’Oliveira Coelho, Robert L. Anemone, Susana Carvalho

**Affiliations:** 1 University of Oxford, Primate Models for Behavioural Evolution Lab, Institute of Human Sciences, Oxford, United Kingdom; 2 Universidade de Coimbra, Centre for Functional Ecology (CFE), Coimbra, Portugal; 3 University of North Carolina at Greensboro, Department of Anthropology, Greensboro, North Carolina, United States of America; 4 Universidade do Algarve, Interdisciplinary Centre for Archaeology and Evolution of Human Behaviour (ICArEHB), Faro, Portugal; 5 Gorongosa National Park, Sofala, Mozambique

**Keywords:** Geospatial Paleontology, Southeast Africa, Late Miocene, Remote Sensing, Unsupervised Learning

## Abstract

**Background:**

Paleoanthropological research focus still devotes most resources to areas generally known to be fossil rich instead of a strategy that first maps and identifies possible fossil sites in a given region. This leads to the paradoxical task of planning paleontological campaigns without knowing the true extent and likely potential of each fossil site and, hence, how to optimize the investment of time and resources. Yet to answer key questions in hominin evolution, paleoanthropologists must engage in fieldwork that targets substantial temporal and geographical gaps in the fossil record. How can the risk of potentially unsuccessful surveys be minimized, while maximizing the potential for successful surveys?

**Methods:**

Here we present a simple and effective solution for finding fossil sites based on clustering by unsupervised learning of satellite images with the *k*-means algorithm and pioneer its testing in the Urema Rift, the southern termination of the East African Rift System (EARS). We focus on a relatively unknown time period critical for understanding African apes and early hominin evolution, the early part of the late Miocene, in an overlooked area of southeastern Africa, in Gorongosa National Park, Mozambique. This clustering approach highlighted priority targets for prospecting that represented only 4.49% of the total area analysed.

**Results:**

Applying this method, four new fossil sites were discovered in the area, and results show an 85% accuracy in a binary classification. This indicates the high potential of a remote sensing tool for exploratory paleontological surveys by enhancing the discovery of productive fossiliferous deposits. The relative importance of spectral bands for clustering was also determined using the random forest algorithm, and near-infrared was the most important variable for fossil site detection, followed by other infrared variables. Bands in the visible spectrum performed the worst and are not likely indicators of fossil sites.

**Discussion:**

We show that unsupervised learning is a useful tool for locating new fossil sites in relatively unexplored regions. Additionally, it can be used to target specific gaps in the fossil record and to increase the sample of fossil sites. In Gorongosa, the discovery of the first estuarine coastal forests of the EARS fills an important paleobiogeographic gap of Africa. These new sites will be key for testing hypotheses of primate evolution in such environmental settings.

## Introduction

**Figure 1:**
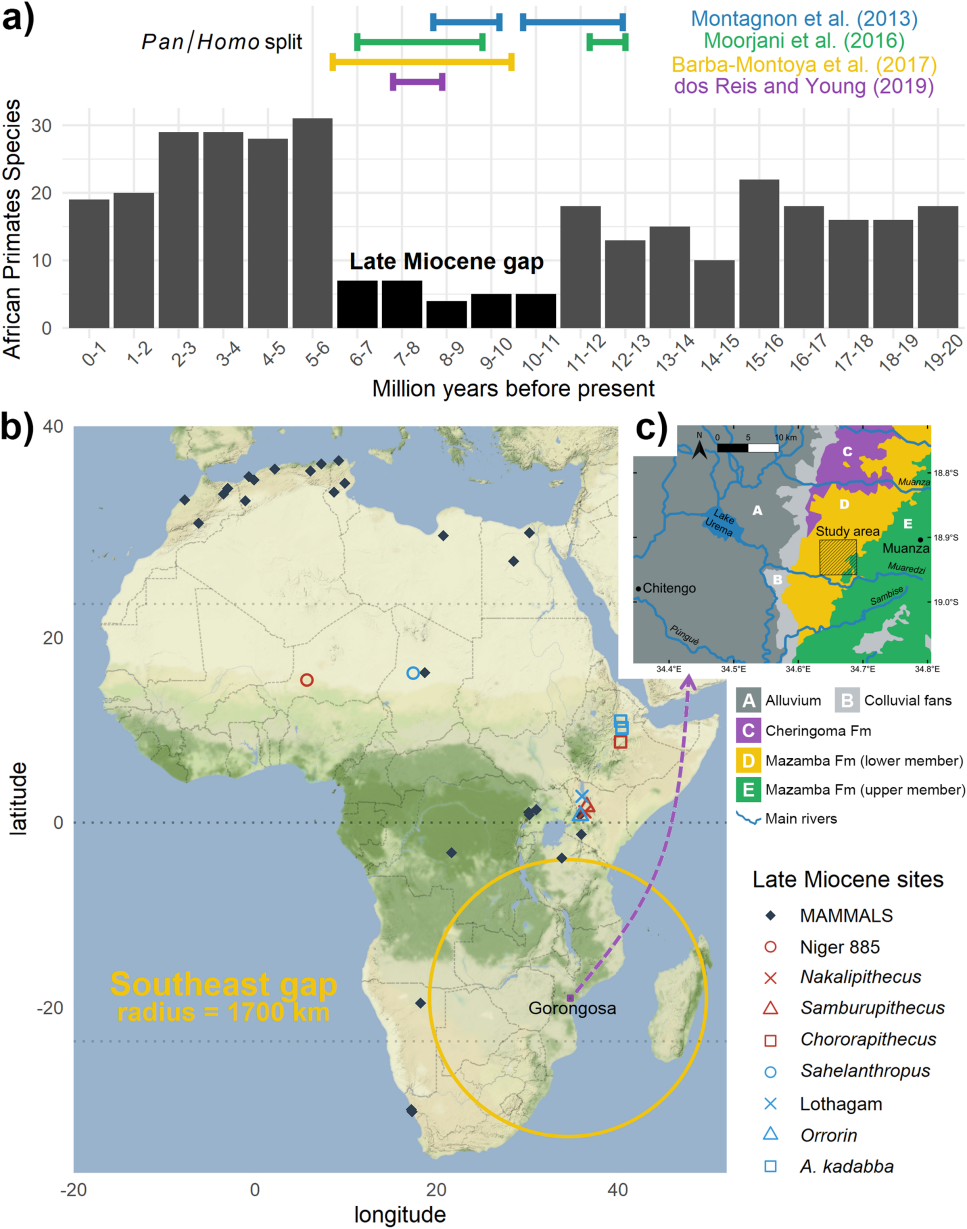
The great gaps of the African late Miocene: (A) Time gap**: **during this key period the African fossil record of primates is very incomplete (evaluated through species richness); but notice the split estimates from genomics; (B) spatial gap: virtually no fossils of this age are known in southeastern Africa, notice the strategic location of Gorongosa. Data extracted from paleobiodb.org, map adapted from Bobe et al. (2018); (C) study area for *k*-means within the geological context of Gorongosa, adapted from Habermann et al. (2019).

Paleontological and molecular evidence indicate that *Homo* shared a most recent common ancestor (MRCA) with the *Pan* lineage during the late Miocene (11.6–5.3 Ma) in Africa (Montagnon, 2013; Moorjani et al., 2016; Barba-Montoya, Dos Reis & Yang, 2017; Dos Reis & Yang, 2019). This makes this period critical to understand the origins of our clade, and yet the geographic distribution of African fossil sites during this time is surprisingly sparse and strongly biased (Figs. 1A, 1B). Currently only three regions contain lithostratigraphic units that have yielded possible early hominins from the late Miocene: Djourab Desert, Chad (*Sahelanthropus tchadensis* Brunet et al., 2002); Tugen Hills, Kenya *(Orrorin tugenensis* Senut et al., 2001); and Middle Awash, Ethiopia (*Ardipithecus kadabba* Haile-Selassie, 2001).

If we expand this sample to include fossils of the African great apes during the same time period, the record is likewise sparse (Andrews, 2019), being limited to the gorilla-like *Chororapithecus abyssinicus* from Ethiopia (Suwa et al., 2007), *Samburupithecus kiptalami* (Ishida & Pickford, 1997) and the basal hominid *Nakalipithecus nakayamai* (Kunimatsu et al., 2007) from Kenya, plus some taxonomically uncertain dental and mandibular finds from Kenya and Niger (Table 1). Furthermore, there is still no consensus regarding the phylogenetic relationships among late Miocene African hominids or how they relate to the MRCA (Senut, 2010, 2015; Kunimatsu et al., 2016). Even less understood are the biogeographic distributions and temporal spans of these taxa that existed within or around the estimated time range for the *Pan/Homo* divergence event. For the great apes, clear fossil evidence only reappears much more recently, in the Pleistocene, demonstrating the temporal extension of these gaps in the fossil record (McBrearty & Jablonski, 2005).

**Table 1:** All hominids described for Africa during the Late Miocene (11.6–5.3 Ma).

Age (Ma)	Fossil site(s)		Taxon	Reference(s)
ca. 5.4	ESC2;3;8, Gona, Ethiopia		*Ardipithecus kadabba*	(Simpson et al., 2015)
5.6–5.2*	Amba East, Ethiopia		*Ardipithecus* cf. *kadabba*	(Haile-Selassie, 2001; Haile-Selassie, Suwa & White, 2004)
5.77–5.54	ALA; ASK; DID; STD^†^		*Ardipithecus kadabba*	
5.8–5.7	Tugen Hills, Kenya	Kapcheberek	*Orrorin tugenensis*	(Senut et al., 2001; Sawada et al., 2002; Pickford & Senut, 2005; Senut, 2010, 2015; Senut, Pickford & Gommery, 2018)
5.9–5.8		Kapsomin	*Orrorin tugenensis*	
			*Incertae sedis* (early *Gorilla?*)	
ca. 6.1		Cheboit	*Orrorin tugenensis*	
			*Incertae sedis* (early *Pan*?)	
		Aragai	*Orrorin tugenensis*	
6–5	Nkondo, Uganda		cf. Gorillini	(Pickford et al., 1988)
6.3	ABD1, Gona, Ethiopia		cf. *Ardipithecus kadabba*	(Simpson et al., 2015)
6.5–5^‡^	Lothagam, Kenya		Homininae indet.	(Leakey & Walker, 2003)
7.34-7.1^§^	Toros-Menalla, Chad		*Sahelanthropus tchadensis*	(Brunet et al., 2002, 2005)
8.0	Ch’orora, Ethiopia		*Chororapithecus abyssinicus*	(Suwa et al., 2007)
9.5	Samburu Hills, Kenya		*Samburupithecus kiptalami*	(Ishida & Pickford, 1997)
9.9–9.8	NA39, Nakali, Kenya		*Nakalipithecus nakayamai*	(Kunimatsu et al., 2007)
			Hominidea indet.	(Kunimatsu et al., 2016)
11–8^¶^	N 885, Niger		*Incertae sedis* (early *Pan*?)	(Pickford et al., 2008, 2009)

**Notes:**

* Probably closer to 5.2 Ma than 5.6, but the Kuserale Mb of the Sagantole Fm is bracketed as in Renne et al. (1999).

† Fossiliferous localities in the Asa Koma Mb of the Adu Asa Fm (Middle Awash, Ethiopia). ALA = Alayla (ALA-VP-2), ASK = Asa Koma (ASK-VP-3), DID = Digiba Dora (DID-VP-1); STD = Saitune Dora (STD-VP-2).

‡ Not the KNM-LT 329 mandible, but two teeth (KNM-LT 22930 M_3_; KNM-LT 25935 I_1_) from older deposits (Upper Nawata).

§ Cosmogenic nuclide dating (Lebatard et al., 2010).

¶ Only biostratigraphic dating available, a more conservative range would be 11.6–5.3 Ma (Late Miocene).

Fundamentally, many of these uncertainties originate from significant gaps in the fossil record. As a discipline, paleoanthropology seeks to answer key questions regarding origins and adaptations of our and other related lineages, but the available data are often too sparse to provide satisfactory answers. Overcoming these limitations may depend largely on developing new strategies for field work, targeting areas and time periods that are undersampled and poorly understood. This may involve considerable risk for paleoanthropological campaigns that most field workers and funding institutions might not be willing to take (Emerson & Anemone, 2012). Moreover, finding hominins is not a straightforward endeavour. For instance, it took the Leakeys 33 years of work at Olduvai Gorge to find their first hominin, the holotype skull of *Paranthropus boisei* (Leakey, 1959). Therefore, innovative risk-aversion methods that can maximize information and improve current surveying approaches are urgently needed (Njau & Hlusko, 2010; Anemone, Emerson & Conroy, 2011).

**Figure 2:**
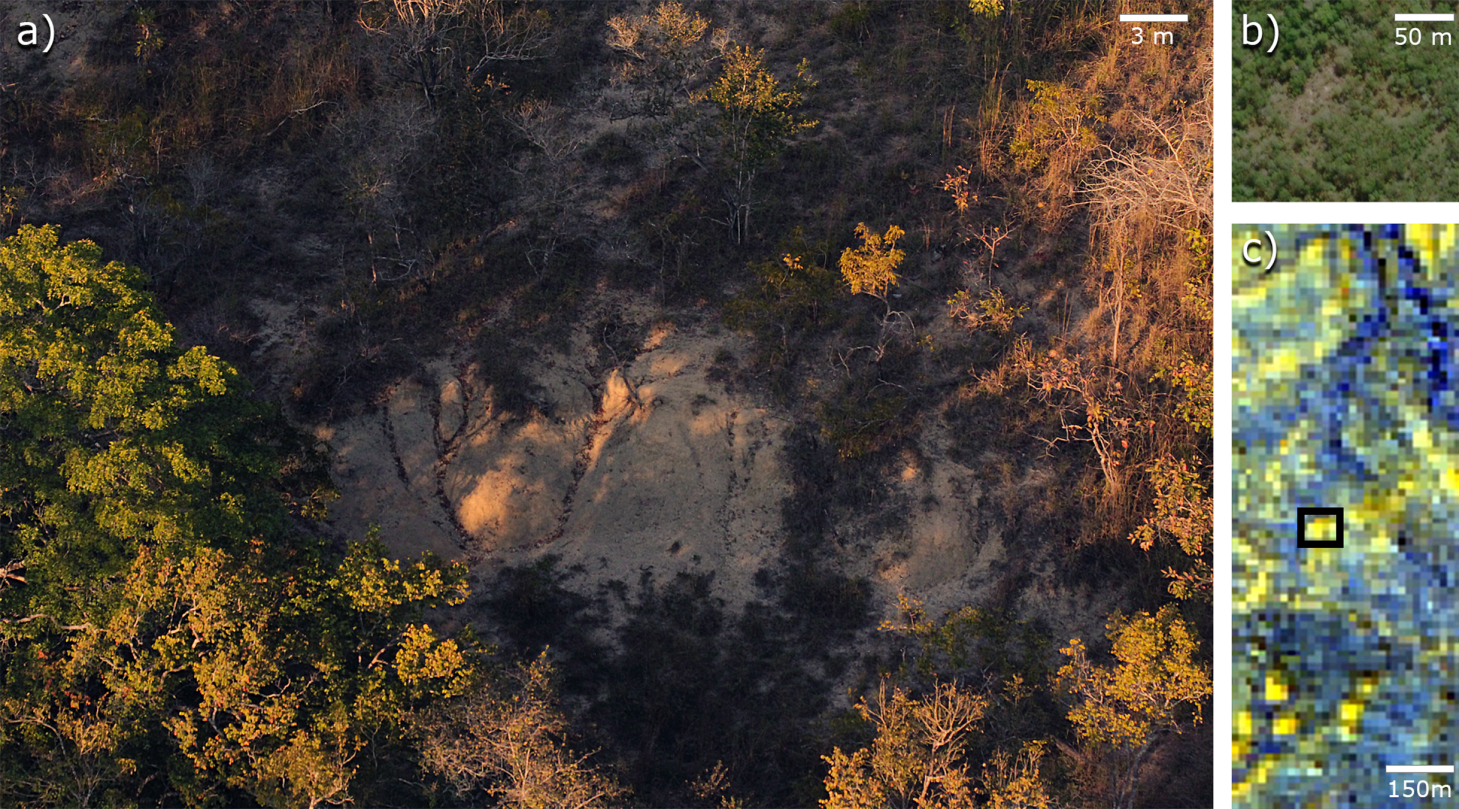
The miombo woodland and the challenges it presents to fossil prospecting: Gorongosa Paleontological Locality 1 (GPL-1). (A) GPL-1 outcrops, notice how the surrounding vegetation is far more dense and extensive than in typical fossil sites from the EARS; (B) GPL-1 in high-resolution satellite image, extracted from bing.com, shows a reduction of vegetation, but outcrops are barely noticeable; (C) GPL-1, in a black rectangle, appears brighter than surrounding areas, when being mapped by lower resolution Landsat 8 false colour (infrared) image, and the same happens with other fossil sites, suggesting that infrared bands might be a useful indicator of fossiliferous deposits.

**Figure 3:**
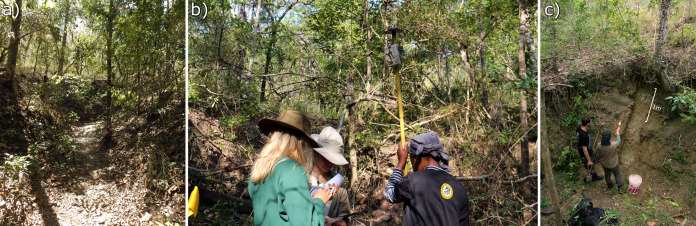
Surveying for fossils in a densely vegetated context. (A) Despite the ground foliage and dense vegetation, in situ and surface evidence of fossils abound in the gully valley connecting GPL-12 to GPL-12B; (B) Systematic mapping and collecting of surface fossil finds by students of the field school; (C) Side gully (~3 m deep) exposure and shovel test pit at GPL-12. Photographs are from the Paleo-Primate Project Gorongosa archive.

How do we know where to look for fossils in unexplored and remote regions? When an area has been extensively surveyed, we can focus on previous work to guide our efforts. If that is not the case, we can use the geology and topography to enhance surveying efforts (Asfaw et al., 1990). However, that is not always possible if the modern topographic and ecological conditions for looking for fossil are not ideal. In places like the miombo woodlands of Gorongosa (Fig. 2), where nine late Miocene paleontological localities have recently been described, the surrounding dense vegetation cover can make reading the landscape for clues to fossiliferous deposits extremely difficult (Habermann et al., 2019). The fossil sites are found just north of the Muaredzi river and east of Lake Urema, between the villages of Chitengo and Muanza (Fig. 1C). The terrain of the study area can be described as flat to slightly undulating, with active incision and upstream erosion of stream sources. Topographic relief is also dotted by island thickets on termitaria hills. The vegetation in the study area is dominated by closed canopy *Brachystegia* (miombo) woodlands, with riverine forests occurring along the drainage network (Fig. 3). The grass layer density can vary greatly spatially and across the seasons, from widely spaced grass tufts in sandier substrates, to medium and tall (>2 m height) grass layers that are subject to regular, typically annual, fires (Tinley, 1977).

Modern geospatial technologies such as remote sensing, handheld GPS devices and geographic information systems (GIS) software have been applied to different paleoanthropological research questions with spatial components (Conroy, 2006; Egeland, Nicholson & Gasparian, 2010; Anemone & Conroy, 2018). Surveys aimed at discovering new fossil assemblages with hominins have also been guided by military, geological and topographic maps, aerial photography and satellite images (Asfaw et al., 1990; Malakhov, Dyke & King, 2009; Hlusko, 2018; Habermann et al., 2019). With recent advances in artificial intelligence, cheaply available computer power, and free access to satellite imagery of reasonable resolution, paleoanthropologists and GIS technicians have been implementing machine learning techniques to automate the demanding visual analysis of remote fossil site detection (e.g., Anemone, Emerson & Conroy, 2011; Conroy et al., 2012, 2018; Conroy, 2014; Emerson et al., 2015; Block et al., 2016; Wills, Choiniere & Barrett, 2018).

Here, we pioneer the application of such automated computational approaches for remote fossil site detection within the EARS (East African Rift System), specifically at its youngest and southernmost subsection, the Urema Rift (where rifting initiated at ~3 ± 1 Ma), in Gorongosa National Park, Mozambique (Böhme et al., 2006; Steinbruch, 2010; Fonseca et al., 2014; Macgregor, 2015). The southern part of the EARS is far less explored than its northern counterpart, which is rich in lacustrine and fluvial paleontological settings (Bobe et al., in press). The first fossil sites of the Urema Rift and their geological features have been recently described by Habermann et al. (2019). All the sites described are part of the lower member of the Mazamba Formation which has been attributed to a Miocene or Mio-Pliocene age (Real, 1966; Grantham et al., 2011; Pickford, 2013; Habermann et al., 2019). Preliminary authigenic ^10^Be/^9^Be dating suggests the fossil sites fit into the earliest part of the late Miocene interval (Bobe et al., 2021). The lower Mazamba successions record coastal nearshore conditions that formed in a shallow ramp setting by marine transgression that occurred prior to rifting. North-eastern sites tend to be richer in fossils of marine fauna and are dominated by sand, while the southern sites are dominated by basal conglomeratic and sandy units overlain by clayey sandstones and sandy marl- and claystone facies (Habermann et al., 2019). The late Miocene paleoenvironments of Gorongosa were reconstructed as estuarine coastal forests and woodlands (Habermann et al., 2019)—a unique context in the EARS—that is both promising for the presence of primates (Nowak, Barnett & Matsuda, 2019) and crucial for testing biogeographic hypothesis of early hominin evolution in estuarine or deltaic wetlands (Wrangham, 2005) and in coastal forest biomes of eastern Africa (Kingdon, 2003; Joordens et al., 2009, 2019; Bobe, Martínez & Carvalho, 2020).

In order to find new fossil sites in Gorongosa, a decision support system based on unsupervised learning of satellite images was created to guide prospecting during the 2018 field season. This was achieved by applying a simple clustering algorithm to satellite images of the field area. There are only two other examples in the literature that applied unsupervised clustering for detecting fossil sites, one in the Uinta Basin, Utah (Conroy, 2014) and the other in the Bighorn and Great Divide Basins, Wyoming (Conroy et al., 2018). Cluster analysis does not use categories that tag objects with prior identifiers (class labels). The absence of categorical information distinguishes data clustering (unsupervised learning) from classification or discriminant analysis (supervised learning). The aim of clustering is to find structure in data and it is therefore exploratory in nature (MacQueen, 1967). One of the most popular and simple clustering algorithms, $k$-means, was first conceptualized in the 1950s (Steinhaus, 1956) and while thousands of different clustering algorithms have been published since then, $k$-means is still widely used because of its effectiveness, speed and simple implementation (Bock, 2008). While $k$-means is a standard technique for many remote sensing applications, it has never been used for fossil site detection. Here, for the first time, this algorithm has been adopted for the purpose of finding fossil sites using satellite imagery.

The goal of this pilot study was to determine if $k$-means, a simple unsupervised computer vision technique for processing satellite images, could improve our ability to identify fossil sites in a limited region of interest in Gorongosa with no a priori knowledge of the geology, stratigraphy, topography and land cover. In addition, we sought to characterize the lower Mazamba Formation (see Habermann et al., 2019) through extensive ground surveys to catalogue the presence and distribution of paleontological resources. These new fossil discoveries will help us to better understand an ancient ecosystem that existed during a crucial period of African great ape diversification and hominin origins. The insights gained from our fieldwork, in combination with the analyses of satellite imagery, allow us to gauge the accuracy of our model and to refine future iterations of these statistical modelling approaches for fossil site detection.

## Materials & methods

**Figure 4:**
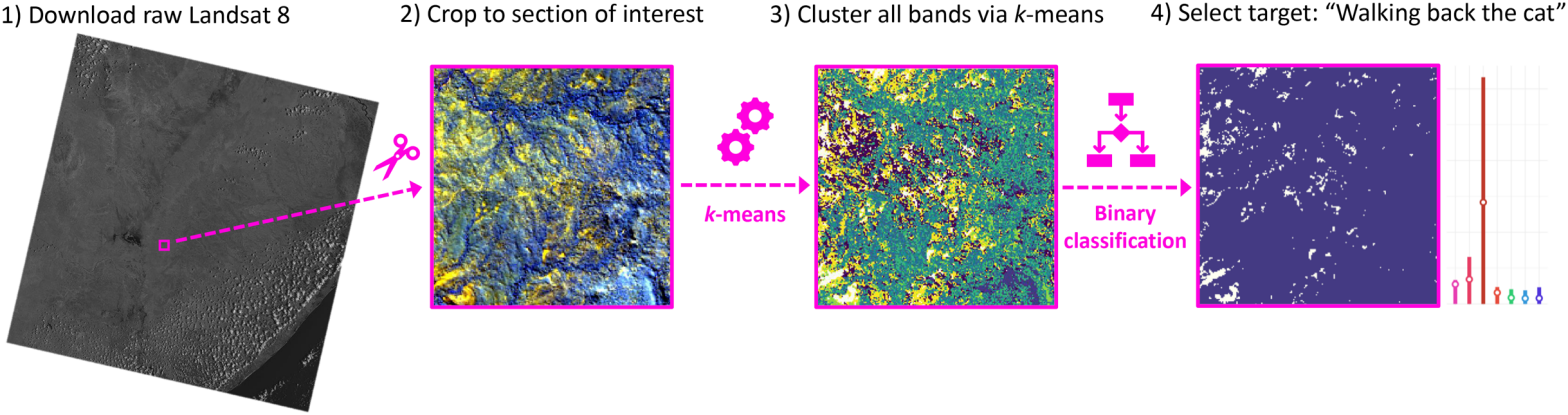
Flowchart of the algorithmic pipeline used for remote fossil site detection. (1) Example of one of the seven spectral bands satellite images used in this study; (2) false colour map based on the infrared bands, after cropping to study area; (3) results of clustering using all seven spectral bands; (4) Binarize clusters for classification by selecting the cluster that contains most fossil sites as the target class (“walking back the cat”) versus all other clusters combined into a single class.

Field experiments were approved by Dr. Marc Stalmans, Director of the Department of Scientific Services of Gorongosa National Park in Mozambique (project number: PNG/DSCi/C019/2018). The code for the complete protocol and input files are available in an open access repository at https://github.com/Delvis/kmeansGorongosa/. The dataset consists of a freely available atmospherically corrected scene with 30 × 30 m resolution of Landsat 8 OLI (Roy et al., 2014) from July 28^th^, 2017 (id: LC08_L1TP_167073_20170628_20170714_01_T1), covering a portion of central Mozambique. Spectral data from Landsat and other medium-resolution satellites is updated daily and can be accessed at the USGS Earth Explorer website (https://earthexplorer.usgs.gov/). After downloading the satellite image, we applied a cropping filter to restrict it to an area of interest known to contain late Miocene to early Pliocene deposits (Real, 1966; Grantham et al., 2011), corresponding to approximately 36 km^2^, in the lower Mazamba Formation within Gorongosa National Park (Fig. 1C). A multidimensional matrix containing the brightness values for seven spectral bands (two short-wave infrared, one near-infrared, three visible ‘RGB’, and one ultrablue) of Landsat 8 in UTM/WGS 84 coordinate system was processed through $k$-means, an unsupervised learning algorithm that can split the satellite image into different *k* clusters based on the spectral pattern of each pixel (a 30 × 30 m point in space). The clusters were then compared to geocoordinates of known late Miocene fossil sites in Gorongosa (available in Habermann et al., 2019) in order to determine whether there was a consistent visual pattern matching cluster(s) with fossil sites, as in Conroy’s (2014) ‘walking back the cat’ approach. In other words, to algorithmically retrace our steps to identify other potential fossiliferous outcrops. This is achieved by generating clusters in an unsupervised manner, and then selecting the cluster(s) containing most fossil sites as the target cluster(s) for the field season (Fig. 4). The advantages of such approach is that it works: (a) when we only have very few fossil sites known in the region; (b) in the absence of any prior knowledge of landscape cover over the proposed survey area; and (c) without pre-processing or training any landcover classes (Conroy, 2014; Conroy et al., 2018). For data analysis purposes, we define “fossil site” as the 30 × 30 m pixel in the Landsat grid that includes the geographic centroid corresponding to a locality in the landscape with exposed outcrops containing either fossilized vertebrates, invertebrates and/or wood reported in the study area (Pickford, 2012, 2013; Habermann et al., 2019).

### Applying the *k*-means algorithm to satellite images

Partitional clustering is a family of unsupervised learning techniques for grouping a set of data points (instances) into $k$ disjoint groups, better known as clusters. The goal is to increase intra-cluster similarity (in this case, Euclidean distance between grouped instances) while decreasing inter-cluster similarity. More specifically, the *k*-means algorithm approximates the best division of $n$ data points in $k$ groups, so that the total distance between each grouped instance ${x_i},\; \; i \in \left\{ {1, \ldots ,n} \right\}$ and its corresponding centroid ${\mu_j},\; \; j \in \left\{ {1, \ldots ,k} \right\}$ is minimized. Formally, this partition occurs through minimization of the within-cluster sum of squares (WCSS), defined as

(1) $$WCSS = \; \mathop \sum \limits_{j = 1}^k \mathop \sum \limits_{i = 1}^n ||x_i^j - {\mu _j}||^2$$

where ||*x^j^_i_ − μ_j_*|| calculates the distance between an instance and a cluster’s centroid.

With a rich history starting in the 1950s, the *k*-means algorithm was independently discovered in various scientific fields (Steinhaus, 1956; Ball & Hall, 1965; Forgy, 1965; MacQueen, 1967; Lloyd, 1982). Its most standard implementation can be broken down into two stages: initialization, where $k$ cluster centroids $({\mu _1}, \ldots {\mu _k})$ are randomly selected (Forgy, 1965); and a second, iterative or repeat stage, following Lloyd’s algorithm (Lloyd, 1982). Lloyd’s iterative refinement technique can be further broken down into the assignment and update steps: first, each instance is assigned to the cluster with the closest centroid ${\mu _j}$; then the set of centroids is updated, by recalculating each centroid with the new instances attached to the clusters. This repetition should run iteratively until cluster membership for the entities converges to a stable solution. Computationally, if $C$ and $C$ are the set of centroids obtained at consecutive Lloyd’s iterations, then the algorithm stops when

(2) $$\left| {WCSS\left( C \right) - \; } \right.\left. {WCSS\left( {{C}^{\prime}} \right)} \right| \le \varepsilon ,\; {\rm for \;a \;fixed \;threshold}\; \ll 1$$

The greatest advantage of *k*-means is scalability, as only the centroid coordinates are stored in memory, it can deal with very large datasets. Moreover, every step can be parallelized, which increases computation performance (Wu et al., 2008; Zhao, Ma & He, 2009). Nevertheless, it can be slow to compute, since each instance might be processed many times throughout the iterations. Another limitation is that the results are dependent on the initial random allocation of the centroids (Äyrämö & Kärkkäinen, 2006).

In the specific case of satellite images, each instance is a geolocated point/pixel (30 × 30 m) with associated brightness values for all the seven spectral bands. Therefore, by applying the $k$-means algorithm to such data, each instance is assigned to one of the $k$ clusters, and instances with similar spectral characteristics will cluster together. Thus $k$-means has many uses, including the ability to: (a) explore and mine hidden patterns in the satellite data; (b) partition a dataset into different groups that might correspond to real types of land cover; and (c) feature learning (a data science technique for encoding new features from raw data), since the output clusters can be used as new variables for subsequent modelling approaches (Jain, 2010).

### Clusters as survey guides for fossil site discovery

If we allow a specific cluster, say cluster 1, to be a predictor for fossil sites, after applying a $k$-means where k = 8, we can consider from a statistical classification paradigm: True Positives (TP) = fossil sites on cluster 1; False Positives (FP) = non-localities on cluster 1; False Negatives (FN) = fossil sites on clusters 2 to 8; True Negatives (TN) = non-localities on clusters 2 to 8. The clustering results were validated digitally using geolocated coordinates for fossil sites and non-localities collected through ground surveys during the 2016 and 17 field seasons. Then, the region was revisited during the first two weeks of August 2018 to survey unexplored areas on foot and ground-truth the model. Localities that could not be thoroughly observed due to obstacles such as high vegetation density, dry foliage, difficult access, or other factors, were recorded as NA (not applicable) and were removed from the current analysis. All other surveyed localities were evaluated as either TP, FP, FN or TN and analysed through measures of statistical performance to assess the efficiency of the implemented approach.

To clarify the mechanisms responsible for generating different clusters, a supervised random forest algorithm for classification (Breiman, 2001) was built on top of the new learned features (cluster 1 to 8). Variable importance metrics, like the permuted mean square-error (%IncMSE), can be extracted from the random forest model. Calculating %IncMSE is achieved by internal out-of-bag estimates of error rate, and then verified by reruns excluding each predictor variable (i.e. permutation). For all the trees in the model, the difference between error rates is averaged, and normalized by the standard deviation of the differences, thus calculating a metric of variable importance (Liaw & Wiener, 2002).

## Results

### Digital validation

**Figure 5:**
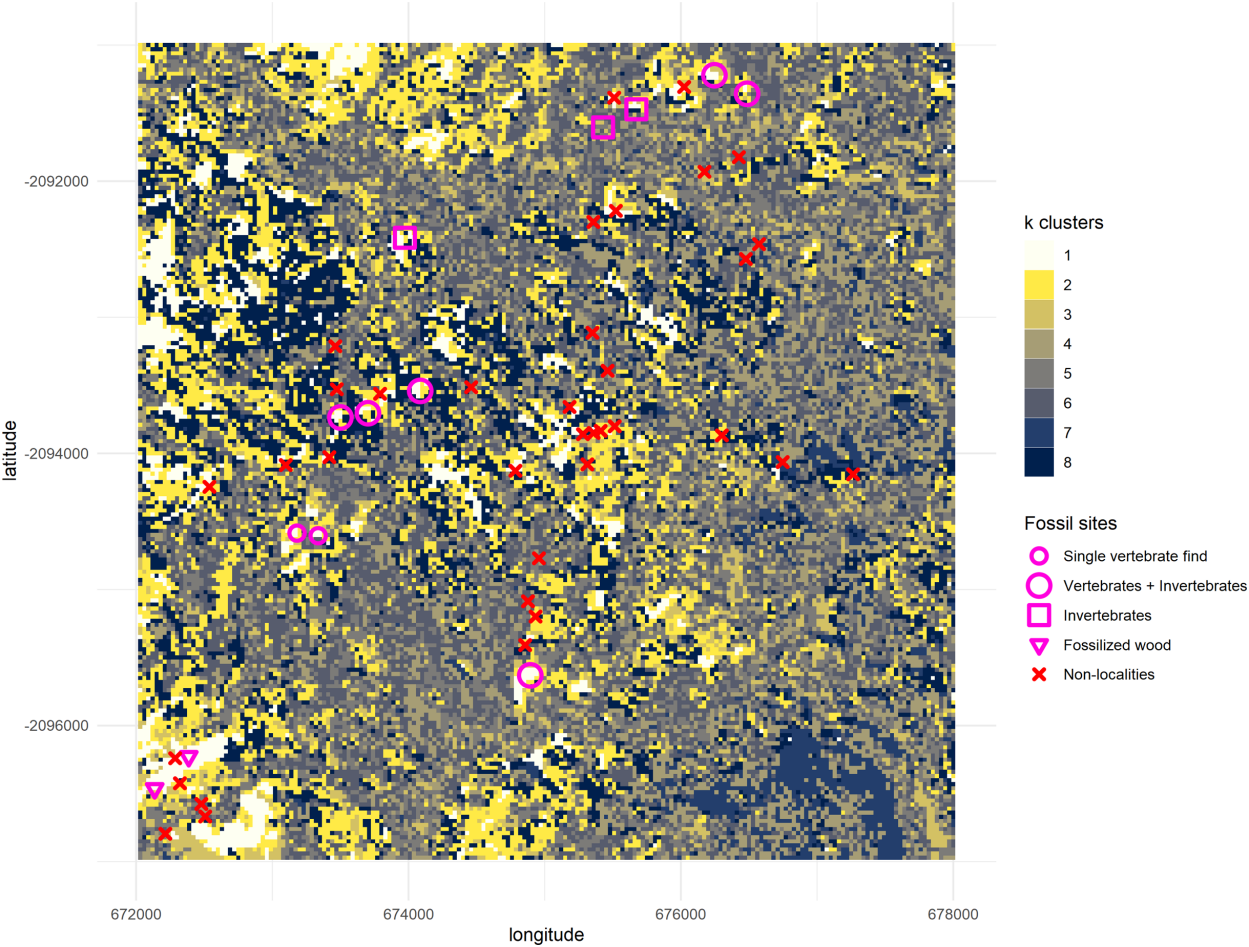
Output from the *k*-means algorithm for data mining. All geolocations “Vertebrates + Invertebrates” and “Invertebrates” recorded by the PaleoPrimate Project Gorongosa team during 2016 and 2017 (Habermann et al., 2019) are plotted over the clusters, as well as the “Single vertebrate find” and “Fossilized wood” localities reported by Pickford (2012, 2013). You can see the cluster 1 (white) tends to be represented in locations with fossil vertebrates, indicating that it has some potential as a new feature/variable for finding new fossil sites. The map displays a 6 by 6 km square; axes scales are in meters.

A plot with the clustering results of the satellite image was rendered and superimposed with a layer representing all the nine known Gorongosa Paleontological Localities (GPL) at the time, provided by Habermann et al. (2019), and two possible vertebrate sites with single-fossil finds of large mammals, plus two sites with fossil wood from earlier work by Pickford (2012, 2013). Additionally, all areas previously surveyed in 2016 and 2017 but without any fossiliferous material discovered (‘non-localities’) were also mapped. Clusters resulting from the $k$-means analysis can thus be compared with all the coordinates to uncover the patterns in the satellite images, such as any combination of spectral wavelength values having more likelihood of remotely detecting the fossil sites (Fig. 5).

**Figure 6:**
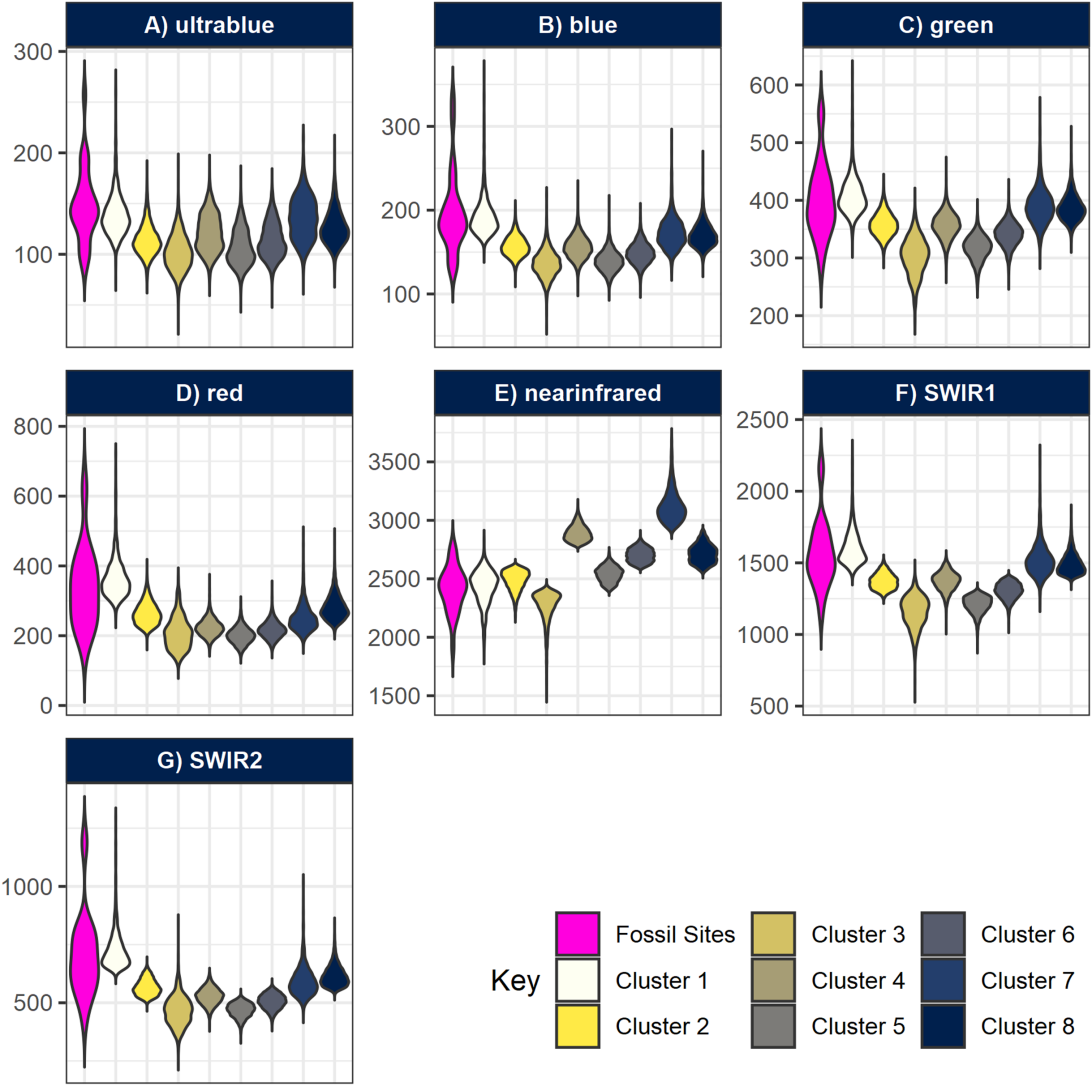
Violin-boxplots comparing sample distribution of spectral bands between clusters. Spectral bands (A) ultrablue; (B) blue; (C) green; (D) red; (E) infrared; (F) short-wavelength infrared 1; and (G) short-wavelength infrared 2 are represented with nine bars comparing the range of spectral bands values at the geocoordinates of the fossil sites, plus the eight clusters generated by *k*-means. Notice how overall, cluster 1 tends to approximate better the true spectral range of known fossil sites in Gorongosa.

All fossil sites containing vertebrate remains recorded in the area overlapped cluster 1 (Fig. 5), as well as the fossil wood sites and two out of three invertebrate sites (only GPL-9 did not match the pattern). However, the non-fossiliferous localities spread randomly over all clusters. Consequently, we decided to consider cluster 1 as a statistical classifier (i.e. predictive cluster) of potential new fossil sites to guide us during survey and thus increase the likelihood of finding sites. As a statistical classifier, cluster 1 cells that do not overlap with a fossil site are considered FP, which is required information to evaluate accuracy and other performance metrics. It should be noted that before being targeted a considerable range of this same cluster remained mostly unexplored, representing only 4.49% of the total area of the cropped image analysed (1,795 pixels out of 40,000). This severe reduction in total space to prospect—from 36 km^2^ to roughly 1.6 km^2^—is one of the main advantages of geospatial paleontology approaches. Prior work has demonstrated that the likelihood of success in locating fossil localities can be greatly increased by highlighting priority targets for prospecting (Oheim, 2007; Egeland, Nicholson & Gasparian, 2010; Anemone, Emerson & Conroy, 2011; Conroy et al., 2018). The spectral range of each cluster generated by $k$-means was also compared with the spectral range of the fossil sites. This again shows us very clearly that cluster 1 matches best the pattern exhibited by the fossiliferous deposits at the lower Mazamba Fm, especially in the NIR, SWIR1, and SWIR2 wavelength regions of the spectral band (Fig. 6).

### Ground-truthing

**Figure 7:**
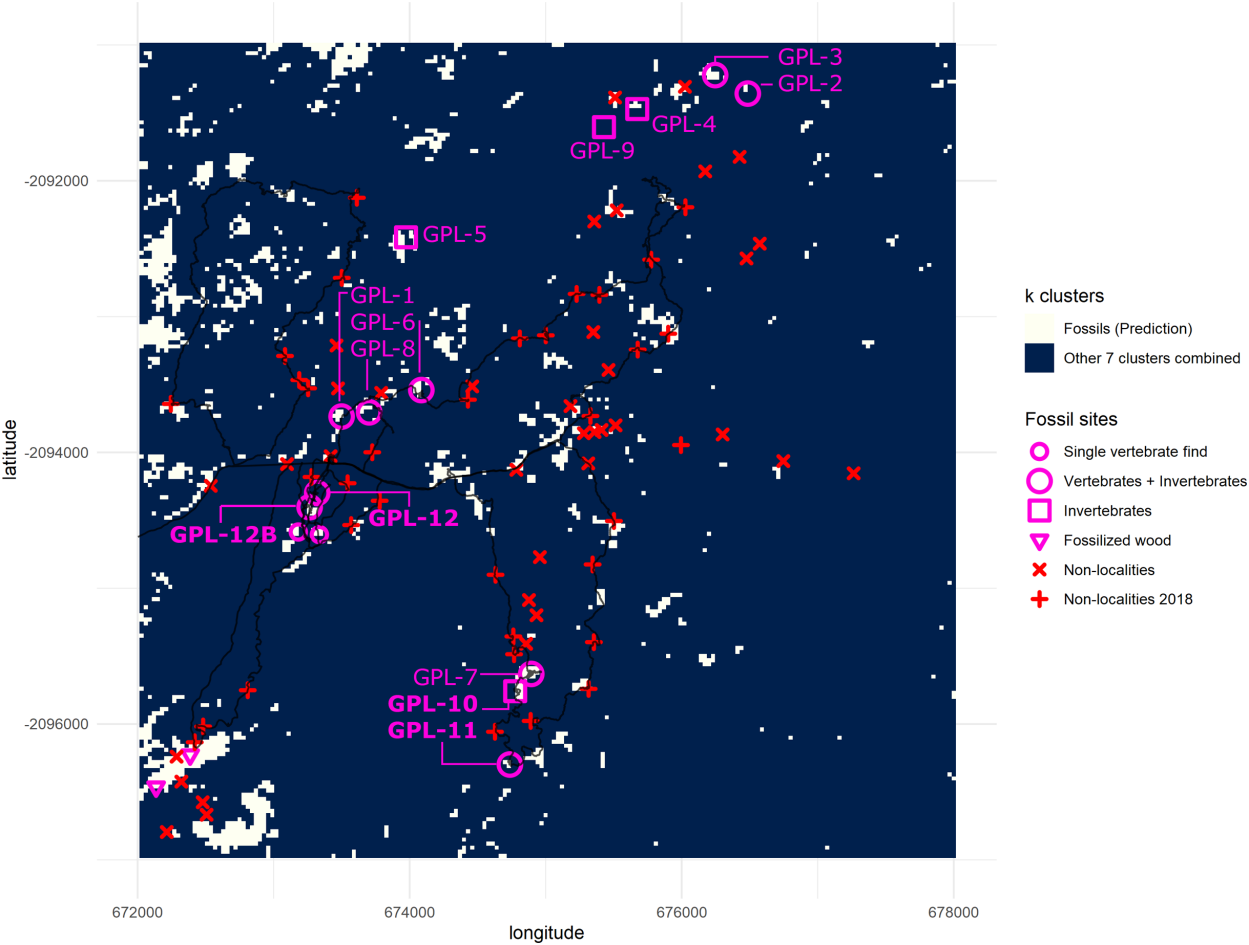
Binarized classification plotting cluster 1 versus all other clusters. New fossil sites GPL-10, 11, 12 and 12B are documented here for the first time. Trackways of surveys during 2018 are drawn in black. Clusters 2–8 are merged into a single cluster and compared against cluster 1 (predictive cluster). Total area = 36 km^2^. One grid square = 1 km^2^. One pixel-cell = 900 m^2^.

During the 2018 season, four new fossil localities were discovered (GPL-10, 11, 12 and 12B), 3 out of 4 completely overlapped with cluster 1, the cluster identified as more likely to enable fossil discovery (Fig. 7). However, the only “miss” (GPL-12, a false negative, since it was not detected by the algorithm), was less than 90 m from a high concentration of white pixels (cluster 1), and thus it was highly likely to be detected following this survey protocol, since it was in the same gully system as GPL-12B—which is detected by $k$-means, and thus was extensively surveyed. Therefore, it can be argued that all the new sites were ultimately discovered by using the $k$-means surveying workflow, including the only one that does not meet the stricter criteria to be considered a true positive, since its discovery was a by-product of surveying guided by the algorithm to that particular area containing a high density of white pixels in proximity, and where GPL-12B was found. There are two possible interpretations, either (1) the buffer areas around a high concentration of cluster 1 pixels might also be more likely to be fossiliferous or (2) the algorithm simply failed to detect this site as the spectral signature differed from other fossil sites. Considering the expressly dense canopy at GPL-12 (Fig. 3), the second is likely the most parsimonious explanation. To be explicit, GPL-9 and -12 were considered false negatives (Table 2A) since cluster 1 of the $k$-means algorithm was defined as the predictive cluster, and it fails to detect them.

**Table 2:** Model goodness-of-fit (A) confusion matrix; (B) performance metrics.

A)		
	Real +	Real −
Predicted +	TP=15	FP=12
Predicted −	FN=2	FP=12

B)		
Measure	Derivations	Value
Sensitivity	*TPR = TP/(TP + FN)*	0.8824
Specificity	*SPC = TN/(FP + TN)*	0.8378
Precision	*PPV = TP/(TP + FP)*	0.5556
Negative predictive value	*NPV = TN/(TN + FN)*	0.9688
Accuracy	*ACC = (TP + TN)/(P + N)*	0.8462

The new outcrops GPL-10 and GPL-11 were located at two river cut-banks (ca. 500 m apart) exposing a series of sandstone beds and they are both rich in invertebrate remains, but vertebrates were also found at the latter. They have been interpreted as representing a coastal delta-plain and fluvio-deltaic to marginal marine conditions, respectively. GPL-12 and GPL-12B are two sites in a gully system that might be continuously exposing fossiliferous sediments from at least three different sandstone layers for more than 100 m (from north to south). These localities yield a remarkably well-preserved fossil record, including in situ and surface finds. Hundreds of identifiable fossil mammals, as well as crocodiles, turtles, and fish, have been recovered from this new area, and it has been tentatively inferred to be a river-dominated estuarine context (Bobe & Carvalho, 2019a, 2019b; Bobe, Martínez & Carvalho, 2020).

Our approach had a high overall accuracy of 84.6%. Statistical measures of performance for model evaluation (defined in Table 2B) such as the negative predictive value (NPV = 96.88%) indicate that the model was able to detect almost perfectly the vast majority of TN ‘non-localities’. In contrast, a low 55.56% precision shows the model overestimated false positives. On the other hand, the model demonstrated a high level of sensitivity (88.24%), because of the very low number of false negatives generated, since only two fossil sites from the database did not match cluster 1 (see Fig. 7). Other metrics like specificity performed well (83.78%), albeit slightly lower than sensitivity, again due to the high number of FP detections, and this performance metric fared much better than the precision metric because it takes into account the model’s ability to correctly detect TN (see Table 2).

**Figure 8:**
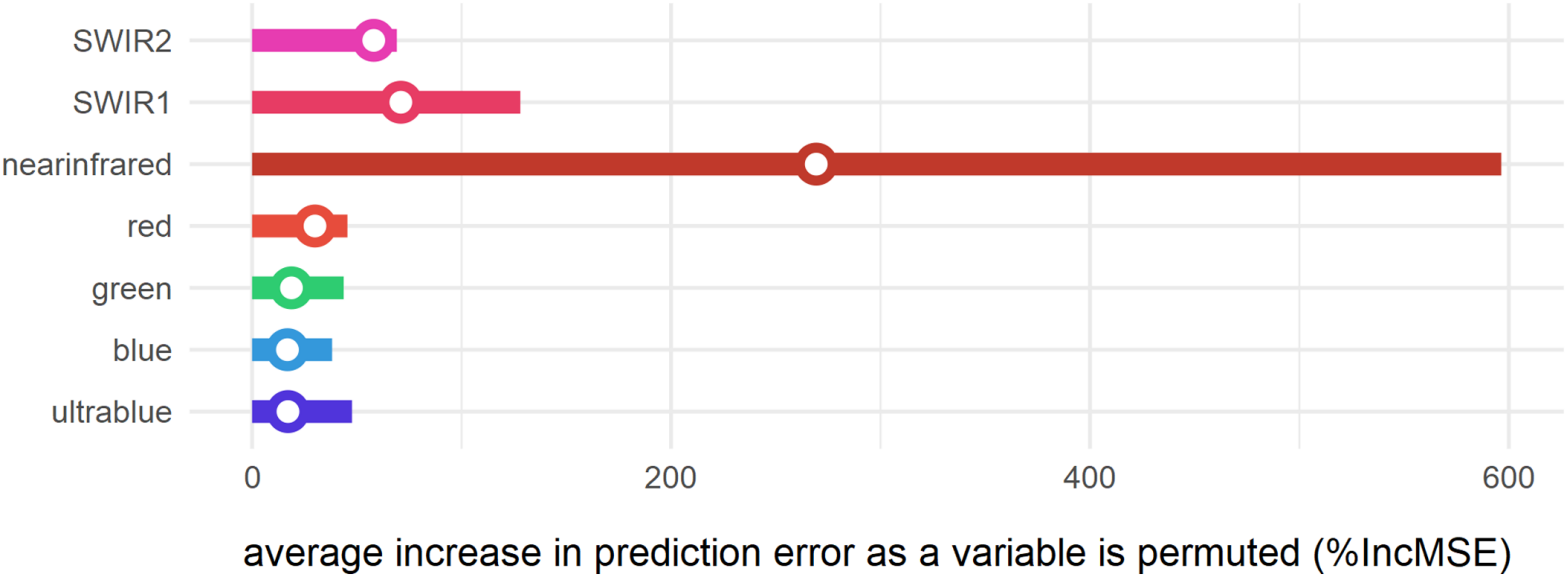
Variable importance of spectral bands for clustering. Bars represent relative importance of the spectral predictors for optimally classifying all clusters as calculated by a supervised random forest algorithm (Breiman, 2001). Specific variable importance for detecting cluster 1 is shown with open circles.

The supervised random forest model (Breiman, 2001; Liaw & Wiener, 2002) was built to assess the relative importance (%IncMSE) of spectral variables in the clustering analysis (Fig. 8). It can thus be demonstrated that for this region of the lower Mazamba Formation the bands in the visible spectrum (0.45–0.67 μm) are the worst predictors of different clusters, while the near-infrared (NIR) is the variable that most contributed to the clustering. This suggests that visual inspection of satellite images (usually depicted in RGB bands) is far from being ideal for this particular task (finding fossil sites). In addition, the bands sampling longer wavelengths in the electromagnetic spectrum (0.85–2.29 μm), from NIR to short-wave infrared 2 (SWIR 2), are notably important at improving detection of cluster 1, and thus are also likely indicators of fossil sites (Fig. 8).

## Discussion

Four new fossil sites from the late Miocene of southerneast Africa have been discovered by remote unsupervised learning. This analysis has also succeeded in pointing out the highest priority regions for future fossil prospecting in the lower Mazamba Formation of Gorongosa. In terms of overall accuracy, our unsupervised approach yielded similar results (84.6%) when compared with other implementations for remote fossil site detection, using supervised neural networks (84.21% with an hold-out sample in Anemone, Emerson & Conroy, 2011; Emerson & Anemone, 2012) and object-based binary classifier (73.1% with an hold-out sample in Emerson et al., 2015). Notice that in an unsupervised pipeline like $k$-means, by definition, all the sample is hold-out as it has not been used to train the algorithm. However, overestimation of false positives (precision = 55.56%) needs to be improved, this can be done in future iterations using different model architectures that also learn from data collected in this campaign. The non-localities are particularly helpful as they can be used as negative weights in models to improve precision.

A limitation of unsupervised methods is that the results are not dependent on any target, since a cluster might not fully match a true cover class. Therefore, a cluster is a subjective entity whose significance and interpretation requires domain knowledge, in this case, the field researchers comparing the different areas targeted by the cluster of interest to understand what they have in common (Jain, 2010). By visually inspecting satellite imagery of higher resolution and having visited multiple points of interest, we suggest that cluster 1 is detecting outcrops with soil erosion, or slightly less vegetated areas that occasionally contain sediment exposures (sometimes hidden under forest canopy). In Gorongosa, the sediment exposures are usually along river valleys or gullies subject to fluvial incision, and the most productive paleontological localities were found at the upstream terminations of tributary channels. In a modern miombo woodland context, like much of Gorongosa, knowing before-hand where to go and find such rare contexts might be sufficient to increase considerably the chances of finding fossiliferous material, simply as a consequence of improved visibility for surveying in the points highlighted remotely through machine learning approaches.

Our variable importance analysis using the random forest algorithm (Breiman, 2001; Liaw & Wiener, 2002) showed the key importance of longer wavelengths for detecting these promising spots within a forest to woodland setting. Infrared reflectance has been used for more than a century to analyse rocks and minerals (e.g. Coblentz, 1906) and its applications in geology are well established (Lyon & Burns, 1963). More recently, near-infrared remote sensing has been applied in soil sciences for multiple purposes, including: mapping proportions of sand, silt, and clay content (Saleh, Belal & Arafat, 2013; Silva et al., 2016); determination of soil salinity (Feyziyev et al., 2016); monitoring soil moisture and capacity of water absorption (Ben-Dor et al., 2002; Whiting, Li & Ustin, 2004); estimate organic carbon content (Hu, Chau & Si, 2015; Viscarra Rossel & Hicks, 2015); differentiate types of clay minerals (Surech, Sreenivas & Sivasamy, 2014); and assessing soil contamination and detection of heavy metals (Mohamed et al., 2016, 2018). Yet, until now, machine learning approaches in geospatial paleontology tended to achieve good results but as “black-boxes” that did not reveal how exactly they were interpreting a “fossiliferous signal”, and thus the relative importance of specific spectral bands for remote fossil site detection has not previously been demonstrated. Here, we show another application of NIR in remote sensing: increasing probability of finding fossils by clustering spectral reflectance values (a proxy for soil properties) that are likely to enhance fossil discovery. While the specific properties are hard to discern at this level of resolution (both spatial and spectral) the most likely candidates are reduced biomass (low foliage and vegetation, are proxies for visibility), and detection by clustering of similar soil types and mineral contents (Mohamed et al., 2018). SWIR1 and SWIR2 variables were also important for the model and higher values are associated with higher soil moisture, and thus are a proxy for water erosion of the soils. This result makes sense considering that most fossil sites in Gorongosa are close to the upstream terminations of the modern drainage system of the region. Erosion proxies have been used before in remote sensing models for fossil site discovery (e.g. Block et al., 2016; Wills, Choiniere & Barrett, 2018). This suggests that the predictive cluster is remotely identifying the same environmental features that Habermann and colleagues observed in the field regarding the “rock exposures most commonly provided by incised gullies at head regions of small tributary channels or by channel flanks, are highly localized due to dense ground vegetation” (2019:727).

Our approach is likely reliable and replicable in other areas of dense vegetation, but it might have limited potential when applied in other areas of the Rift that are drier and have much less vegetation cover and consequently, more exposure. However, previous implementations of unsupervised clustering approaches in modern deserts of North America have been successful at finding Eocene deposits with early primate fauna (Conroy, 2014; Conroy et al., 2018), thus indicating that similar approaches are likely to be useful throughout the EARS. One last issue with $k$-means is that it creates equiprobable clusters, and thus might fail to properly represent the spectral signatures of the landcovers in certain surveying areas, so other unsupervised learning models without this characteristic might also improve the current results (Schubert et al., 2017).

## Conclusions

The $k$-means approach performed well with high overall accuracy, contributing to the discovery of four new fossil sites, albeit with some limitations (see precision metric). To address this constraint, in future field seasons a set of different modelling approaches will be implemented using different architectures and other features besides the spectral bands, such as geo-ecological variables (elevation, slope, aspect, vegetation index, etc.). Importantly, the 2018 field season yielded several new sites as well as numerous negative coordinates (non-localities), and these data are vital to implement supervised approaches, which are more robust and may achieve better results than the method presented here. Considering NIR is usually interpreted as a proxy to biomass content in the soils (Hu, Chau & Si, 2015; Viscarra Rossel & Hicks, 2015), ground-truthing future implementations of different machine learning models should be done in the peak of the dry season in Gorongosa and/or after the wildfires, to further enhance visibility of topographic features, outcrops, and exposures of sedimentary rock. It is still too soon to fully understand how much remote sensing and unsupervised learning methods can be generalized to the problem of remote fossil site detection, but with an accuracy of 84.6% and new fossil sites detected the potential is clear.

What we do know, and what we can know about evolutionary processes is constrained by the fossil record. Indeed, paleontological inference is dependent—as are all historical disciplines—on the contexts that produce the data. The way to avoid biased inferences is thus to find more contexts to sample, so that our data do not increase only in quantity but also in representativeness of the natural and historic processes intricately linked to evolution. Early hominin occupation of humid and watered habitats such as riparian woodlands next to lake margins is well-documented across the basins of the EARS (Cerling et al., 2011; Behrensmeyer & Reed, 2013). But Gorongosa is the first of its kind, a rift valley estuarine and littoral site, partially covered by marine deposits (Habermann et al., 2019). Finding new fossil sites in under-studied African paleo-biomes of the late Miocene, such as coastal forests in estuaries, is crucial in order to test critical hypotheses related to early hominin evolution in such contexts (Kingdon, 2003; Wrangham, 2005; Joordens et al., 2019; Bobe, Martínez & Carvalho, 2020). Future studies should use unsupervised learning algorithms coupled with ground-truthing as these are likely to become key tools to identify areas worth prospecting, leading to discovery of fossil-bearing localities. As these and other computer vision approaches improve, they will directly tackle some of the main limiting factors for paleontological studies: sample size, geographic gaps, and temporal biases, and thereby allowing substantial progress in the pace and content of paleontological and paleoanthropological discoveries.

## Additional Information and Declarations

### Field Study Permissions

The following information was supplied relating to field study approvals (i.e., approving body and any reference numbers): Field experiments were approved by Dr Marc Stalmans, Director of the Department of Scientific Services of Gorongosa National Park in Mozambique (project number: PNG/DSCi/C019/2018).

### Data Availability

The following information was supplied regarding data availability: Files to replicate the kmeans+randomforest pipeline for detecting fossil sites in Gorongosa are available at GitHub: https://github.com/Delvis/kmeansGorongosa. Spectral data from Landsat and other medium-resolution satellites is updated daily and can be accessed at the USGS Earth Explorer website (https://earthexplorer.usgs.gov/).
